# Transportation Optimization with Fuzzy Trapezoidal Numbers Based on Possibility Theory

**DOI:** 10.1371/journal.pone.0105142

**Published:** 2014-08-19

**Authors:** Dayi He, Ran Li, Qi Huang, Ping Lei

**Affiliations:** School of Humanities & Economic Management, Lab of Resources & Environment Management, China University of Geosciences (Beijing), Beijing, P. R. China; Politehnica University of Bucharest, Romania

## Abstract

In this paper, a parametric method is introduced to solve fuzzy transportation problem. Considering that parameters of transportation problem have uncertainties, this paper develops a generalized fuzzy transportation problem with fuzzy supply, demand and cost. For simplicity, these parameters are assumed to be fuzzy trapezoidal numbers. Based on possibility theory and consistent with decision-makers' subjectiveness and practical requirements, the fuzzy transportation problem is transformed to a crisp linear transportation problem by defuzzifying fuzzy constraints and objectives with application of fractile and modality approach. Finally, a numerical example is provided to exemplify the application of fuzzy transportation programming and to verify the validity of the proposed methods.

## Introduction

The transportation problem is a special type of the linear programming problem which arises in many practical applications. And it is one of the earliest and most fruitful applications of linear programming technique. It has been widely studied in Logistics and Operations Management where distribution of goods and commodities from sources to destinations is an important issue. The problem was formalized by the French Mathematician Gaspard Monge in 1781. Tolstoi was one of the first to study the transportation problem mathematically.

In transportation problem the optimal shipping patterns between origins or supply centers and destinations or demand centers should be determined. Suppose that 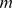 origins are to supply 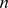 destinations with a certain product. Let 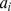 be the amount of the product available at origin 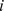, and 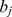 be the amount of the product required at destination 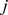. Further, the cost of shipping a unit of product from origin 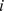 to destination 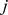 is assumed to be 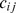, and let 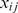 be the amount shipped from origin 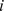 to destination 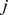. If shipping cost are assumed to be proportional to the amount shipped from each origin to each destination so as to minimize total shipping cost turns out to be a linear programming problem.

In practice, there are many situations where it is impossible to get precise data for the cost parameters, due to complexity and uncertainty of information. A fuzzy transportation problem (FTP) is a transportation problem in which transportation costs, supply and/or demand quantities are fuzzy quantities. Earlier methods for solving fuzzy transportation problems are proposed by [1]–[4]. More recently, Gani and Razak [5] obtained a fuzzy solution for a two stage cost minimizing fuzzy transportation problem in which supplies and demands are trapezoidal fuzzy numbers. Pandian and Natarajan [6] proposed a method, namely fuzzy zero point methods, for finding a fuzzy optimal solution for a fuzzy transportation problem where all parameters are trapezoidal fuzzy numbers. Basirzadeh [7] introduced an approach for solving a wide range of fuzzy transportation problem by ranking fuzzy numbers. Narayanamoorthy et al. [8] proposed a new algorithm called Fuzzy Russell's method for the initial basic feasible solution to a Fuzzy transportation problem. Chaudhuri, et al. [9] present the closed, bounded and nonempty feasible region of the transportation problem using fuzzy trapezoidal numbers.

Most previous research on fuzzy transportation problem assumes that all parameters are fuzzy numbers where the representation is either normal or abnormal, triangular or trapezoidal. Previous reports in literatures typically focus on comparing two or more fuzzy numbers and ranking fuzzy numbers for the purpose of optimization. This approach often achieved a bounded range or nonempty feasible region of transportation quantities and total cost, which are instructive rather than a straightforward solution for decision-makers. Furthermore, in order to effectively solve fuzzy transportation problem, the key factor is the decision-maker's attitude about fuzziness. The possibility theory provides a feasible and scientific tool to describe the decision-maker's attitude about fuzziness. Therefore, we will try to apply possibility theory to solve fuzzy transportation problem. In this paper, we will also consider all parameters in a fuzzy transportation problem as fuzzy numbers. However, we will utilize possibility theory to formalize the fuzzy transportation problem by introducing fractile and modality approach, therefore the decision-maker's attitude about uncertainty can be considered into the decision process, which is of great importance when a decision-maker is confronted with uncertainty or fuzziness.

This paper has five sections. Section 1 is an introduction. In section 2, some basic concepts and possibility theory are introduced. Section 3 is the main part of this paper, where we proposed fuzzy transportation problem and defuzzied it into four crisp linear programming problems by utilizing the fractile or modality approach. In section 4, a numerical example is provided to illustrate the methods developed in this paper. And section 5 concludes.

## Preliminaries

In this section the basic concepts of transportation problem, fuzzy number, trapezoidal fuzzy number, possibility theory and their properties are recalled.

### 2.1 The crisp transportation problem

Mathematically a transportation problem can be stated as follows:
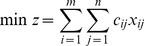
(1)

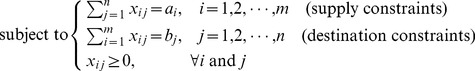
(2)where 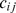 is the cost of transportation of a unit from the 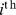 source to the 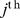 destination, and the quantity 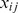 is some non-negative number, which is to be transported from the 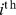 origin to the 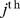 destination. An obvious necessary and sufficient condition for the linear programming problem given in (1) to have a solution is that
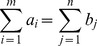
(3)i.e., it is assumed that the total available is equal to the total required. If it is not true, a fictitious origin or destination can be added. It should be noted that the problem has a feasible solution if and only if condition (2) is satisfied. Now the problem is to determine 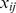, in such a way that the total transportation cost is minimum.

### 2.2 Generalized fuzzy number and its 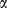-cut

A fuzzy number is an generalization of a regular, real number in the sense that it does not refer to one single value but rather to a connected set of possible values, where each possible value has its own weight between 0 and 1. This weight is called the membership function. A fuzzy number is thus a special case of a convex, normalized fuzzy set of the real line.

Mathematically a generalized fuzzy number 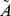, conventionally represented by 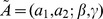, i.e., (left point, right point; left spread, right spread), is a normalized convex fuzzy subset on the real line 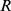 if

i) 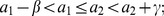


ii) the membership function is of the following form:



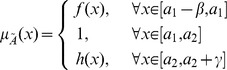
where 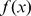 and 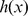 are the monotonic increasing and decreasing functions in 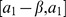 and 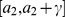 respectively;

iii) 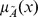 is an upper semi-continuous function;

iv) 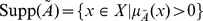 is a closed and bounded interval, i.e., 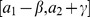.

Specifically an LR-type fuzzy number is obtained from a generalized fuzzy number if the shape functions 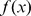 and 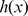 are approximated by 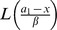 and 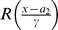 respectively.

The 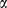-cut of 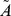 is an interval number denoted by 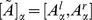, which is explicitly shown for a LR-type fuzzy number, i.e., 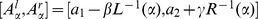 for all 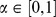.

### 2.3 Trapezoidal fuzzy number

A fuzzy number 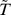 is a trapezoidal fuzzy number denoted by 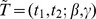 where 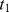, 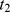, 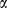, 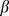 are real number and its membership function 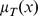 is given below.
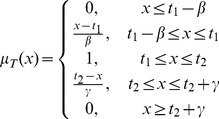



**Figure 1:**
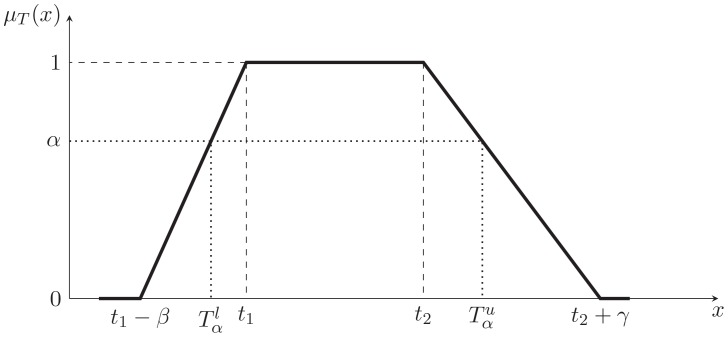
The membership function and 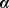-cut of a trapezoidal fuzzy number.

The 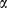-cut of the fuzzy number 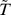 which can be denoted by 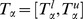, is shown in the Fig. 1.
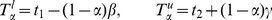



### 2.4 Possibility theory

Possibility theory is an uncertainty theory devoted to handle incomplete information. As such, it complements probability theory. It differs from the latter by using of a pair of dual set-functions (possibility and necessity measures) instead of only one. This feature makes it easier to capture partial ignorance. Besides, it is not additive and makes sense on ordinal structures. The name Theory of Possibility was coined in Negoita and Zadeh [10], inspired by Gaines [11]. In Zadeh's view, possibility distributions were meant to provide a graded semantics to natural language statements. However, possibility and necessity measures can also be the basis of a full-fledged representation of partial belief that parallels probability [12]. Then, it can be seen as a coarse, non-numerical version of probability theory, or as a framework for reasoning with extreme probabilities, or yet as a simple approach to reasoning with imprecise probabilities [13].

If 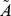 and 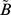 are two fuzzy sets and a fuzzy event 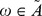, the possibility and necessity of 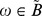 are defined as:
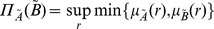


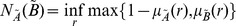



If 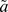 and 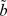 are two fuzzy numbers and their membership function are 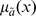 and 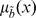 respectively, the measure of possibility and necessity are defined as:
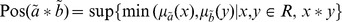


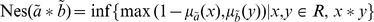



If 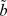 is a crisp number, then the we can achieved that
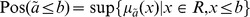


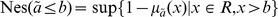



Generally speaking, the possibility degree evaluates to what extent an event is consistent with the knowledge 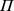, while the necessity degree evaluates to what extent an event is certainly implied by the knowledge.

## Mathematical formulation of fuzzy transportation problem

In this section, we first present the Fuzzy Transportation Problem (FTP). Then, based on possibility theory, treatment of constraints and objective are discussed in order to develop the four formulations of the FTP.

### 3.1 Fuzzy transportation problem

Mathematically the FTP can be described as follows.
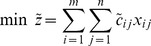
(4)

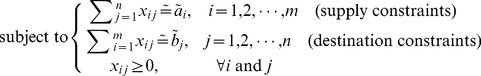
(5)


In the constraints of FTP, the left side is a crisp number while the right side is a fuzzy number. Hence, the constraints produce a fuzzy set. To determine whether a crisp number is in a fuzzy set, the classical method is to use the membership function. If the membership function value of the crisp number is positive, then it can be sure that it belongs to the fuzzy set, and the larger is the value, the higher is the extent. As for the objective function, it is a linear non-negative combination of fuzzy variables. So the objective of FTP is a fuzzy variable too. To minimize the objective, a ranking method of fuzzy numbers should be introduced.

Therefore, in order to solve FTP, we should introduce a measure to determine whether a crisp number belongs to a fuzzy set and a ranking index to rank fuzzy numbers. Generally speaking, let the feasible fuzzy set of the FTP is denoted by 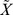, and all possible objective values is in the fuzzy set 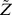. And there is a measure system 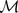 to determine whether a crisp number belongs to a fuzzy set, where 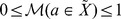. Then the FTP can be described as follows conceptually.
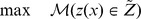


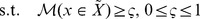
or



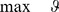


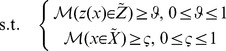
where 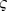 is the degree of 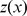 belongs to 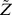 and 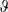 is the degree of 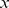 belongs to 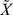.

To be simple, in this paper we use trapezoidal numbers to represent the fuzziness of parameters in FTP, i.e., we assume that
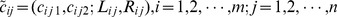


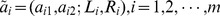


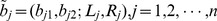



And the possibility and necessity measure are adopted as ranking index.

**Figure 2:**
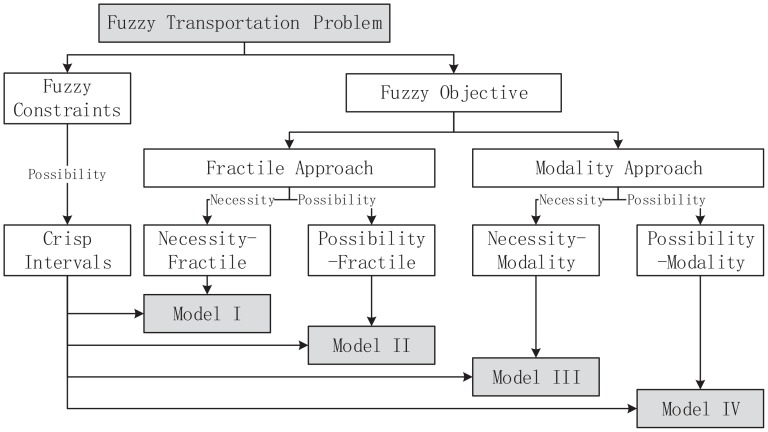
Framework of defuzzifying FTP.

We think that how to solve FTP depends on the decision-maker's attitude to the fuzziness, so it is hard to define which model should be used. Decision-maker can choose any proposed method to solve FTP just according to his attitude to the fuzziness. We propose possibility theory to describe decision-maker's attitude. As FTP consists of fuzzy constraints and objective, this paper deals with them individually as shown in Figure 2. With respect to fuzzy constraints, we utilize possibility theory to convert them into crisp and bounded intervals. As for fuzzy objective, by applying fractile and modality approach we convert it into four formations from the view of necessity and possibility respectively. Then by recombining the converted constraints and objective, four models are established.

### 3.2 Treatment of constraints

As constraints of FTP are equations, and according to the definition of possibility and necessity, we can get
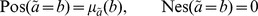



Hence, the treatment of constraints can be considered from the view of possibility exclusively.

It is reasonable and frequently-used that when decision makers are confronted with uncertainty, they can only make sure that the constraints are satisfied to some extent. For the 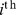 origin 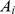 in FTP, the decision-maker expects that the 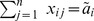 should be satisfied as possible because the production is uncertain. According to the definition of possibility, we can set that the possibility of the constraint be true is larger than a given level 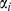, where 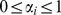, which reflects the extent how the constraint is satisfied.
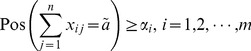
(6)


**Figure 3:**
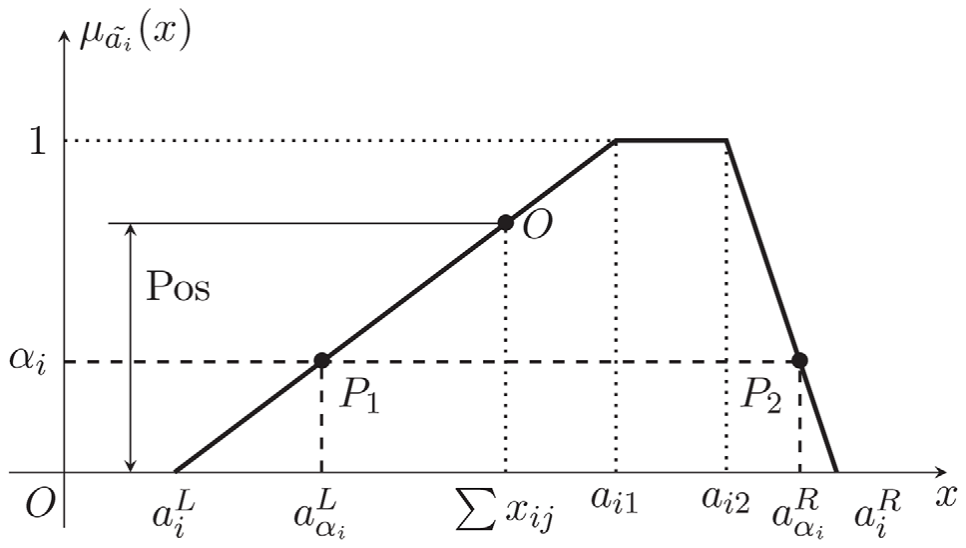
The treatment of constraints.

According to the definition of possibility, the equation (6) is depicted in the Figure 3, where 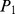 and 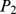 is the left and right bound of 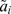
's 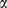-cut.

To ensure the equation (6) can be satisfied, the point 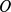 should be above of 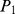 and 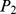, which converts the constraint 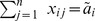 to
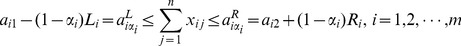
(7)


Similarly, for the 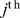 destination 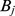, the constraint should be satisfied with the possibility larger than 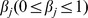, i.e.,
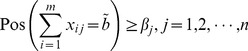
(8)


And it can be converted to
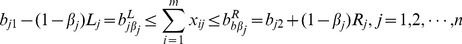
(9)


In such ways, the constraints of FTP are transformed into crisp and bounded intervals.

### 3.3 Treatment of objective

According to the operation algorithm of fuzzy numbers, because the unit cost of FTP is a linear non-negative combination of 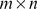 trapezoidal numbers, the objective is a trapezoidal numbers too. Let 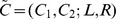 be the objective value. When 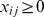, there exists
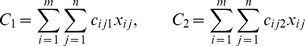


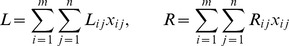



There are two approaches to treat the objective: fractile and modality approach.

#### 3.3.1 Fractile approach

A fractile approach corresponds to the Kataoka's model of a stochastic programming problem [14], [15]. Geoffrion [16] calls the Kataoka's model the fractile criterion approach. By the definition in statistics, 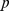-fractile is the value 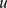 which satisfies
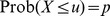
where 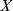 is a random variable. In this definition, 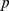-fractile does not generally exist for all 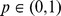. That is why we define 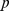-fractile as the smallest value 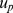 of 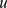 which satisfies



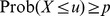



From the viewpoint of Dempster-Shafer theory of evidence [17], it is known that 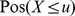 and 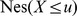 can be regarded as the upper and lower bounds of 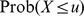 (see [18]). In this sense, 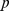-possibility fractile is defined as the smallest value of 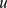 which satisfies
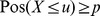
and 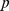-necessity fractile is defined as the smallest value of 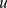 which satisfies



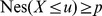



As for the objective of FTP, from the point of necessity it can convert to
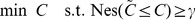
(10)where 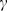 is a parameter to reflect to what extent the decision-maker is certain about the lower bound of the fuzzy event 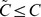. Eq.(10) expresses that the decision-maker minimizes the total cost at a given necessity level.

**Figure 4:**
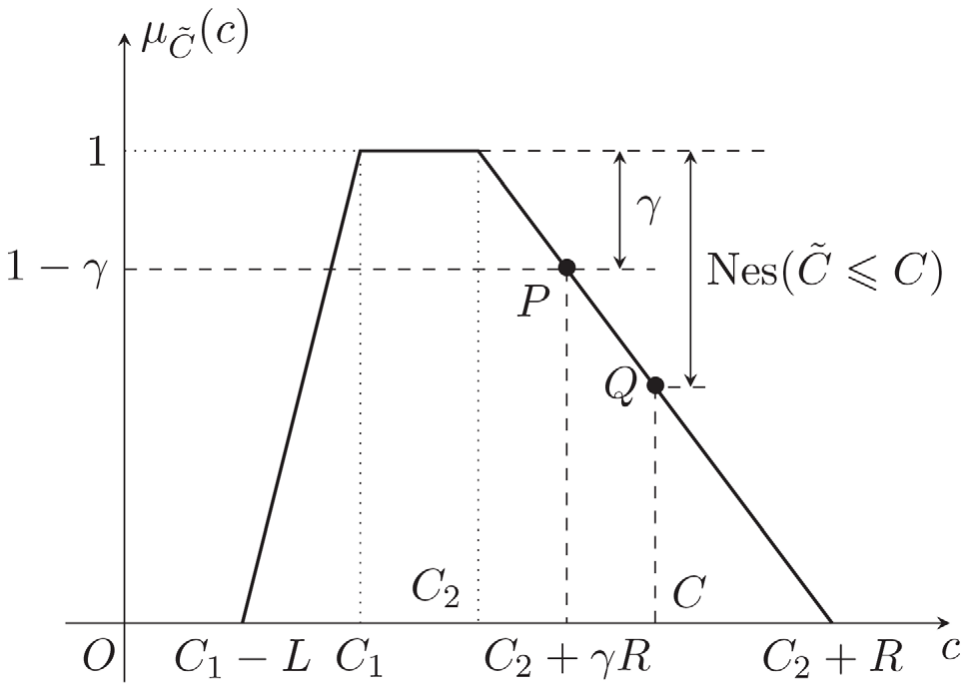
Treatment of objective: necessity-fractile approach.



As shown in Figure 4, we can get
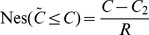
then the constraint in (10) can be converted to

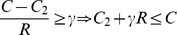



Hence, Eq. (10) turns to
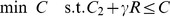
(11)


It is easy to find that the optimal solution of (11) is 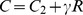. Therefore, the objective function of FTP can be substituted by
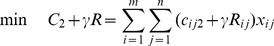
(12)


And with the constraints (7) and (9), the FTP turns to the following model, which is a crisp linear programming problem.
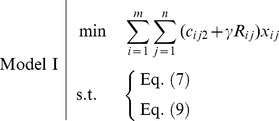



From the point of the possibility and by the fractile approach, the objective of FTP means that
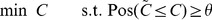
(13)i.e., the decision-maker expects that the total cost should be minimized as the possibility that total cost is not larger than some given level 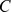.

**Figure 5:**
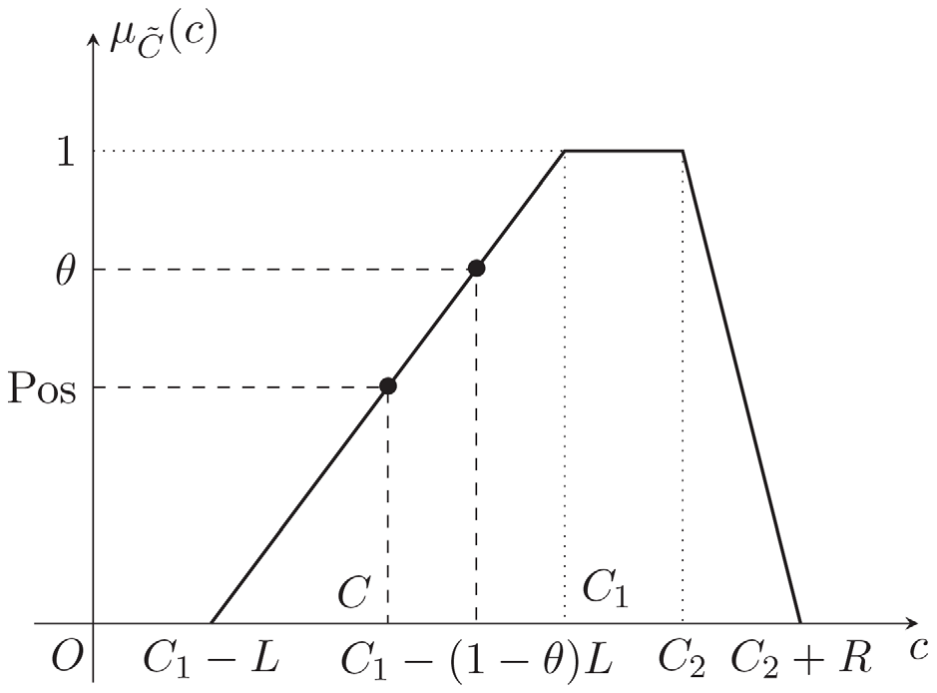
Treatment of objective: possibility-fractile approach.



As shown in Figure 5, we can get
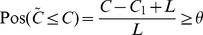
or

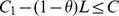



Hence, (13) can be converted to
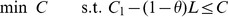
(14)


It is easy to find that the optimal solution of (14) is 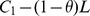, i.e.,
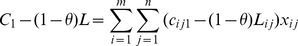
(15)


And with the constraints (7) and (9), the FTP turns to the following model.
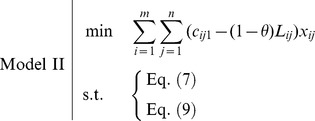



#### 3.3.2 Modality approach

A modality optimization model corresponds to the minimum-risk approach to a stochastic programming problem [15]. The minimum-risk approach is also called the maximum probability approach [14] or the aspiration criterion approach [16]. A modality optimization approach is a dual approach to the fractile optimization one. In this approach, the decision-maker puts more importance on the certainty degree comparing to the fractile approach.

From the point of necessity and modality approach, the decision-maker in FTP expects that the total cost should not be greater than a given level 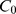, i.e.,
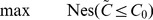
(16)


**Figure 6:**
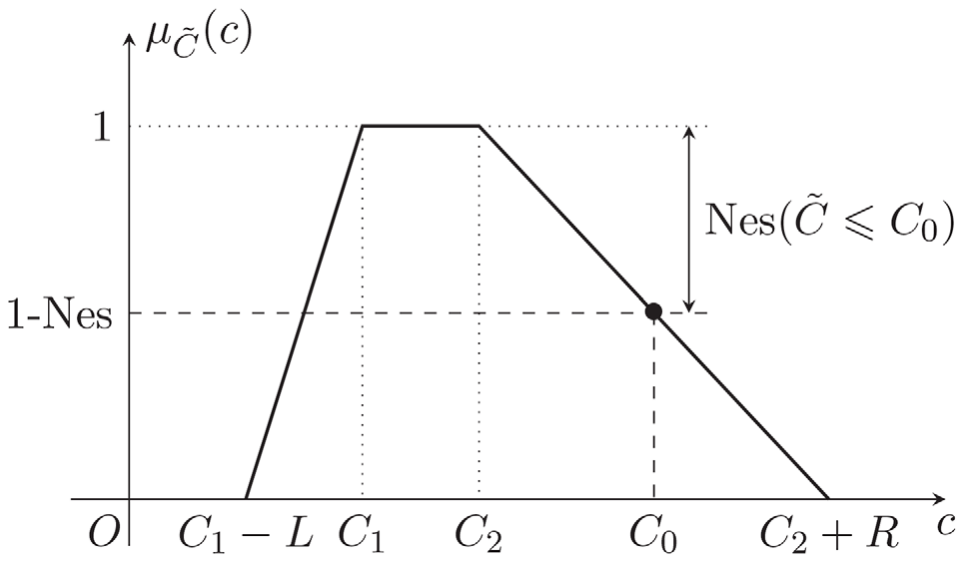
Treatment of objective: necessity-modality approach.

From Figure 6, we can get
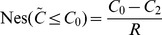
hence, the objective of FTP can be converted to
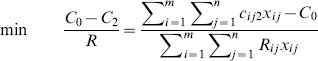
(17)


Such that FTP turns to
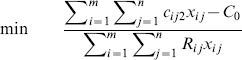


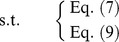



This is a fractional programming problem which can be transformed to a linear programming problem by the substitution
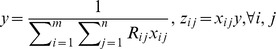



We obtain the following model.
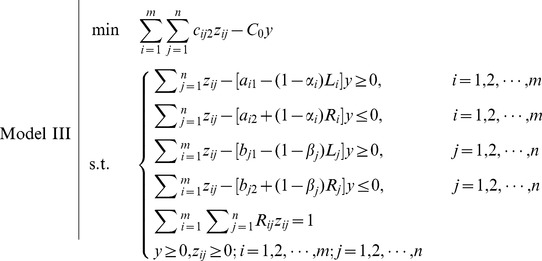



From the point of possibility, the decision-maker expects that the total cost should not be greater than a given level 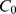, i.e.,
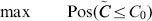
(18)


**Figure 7:**
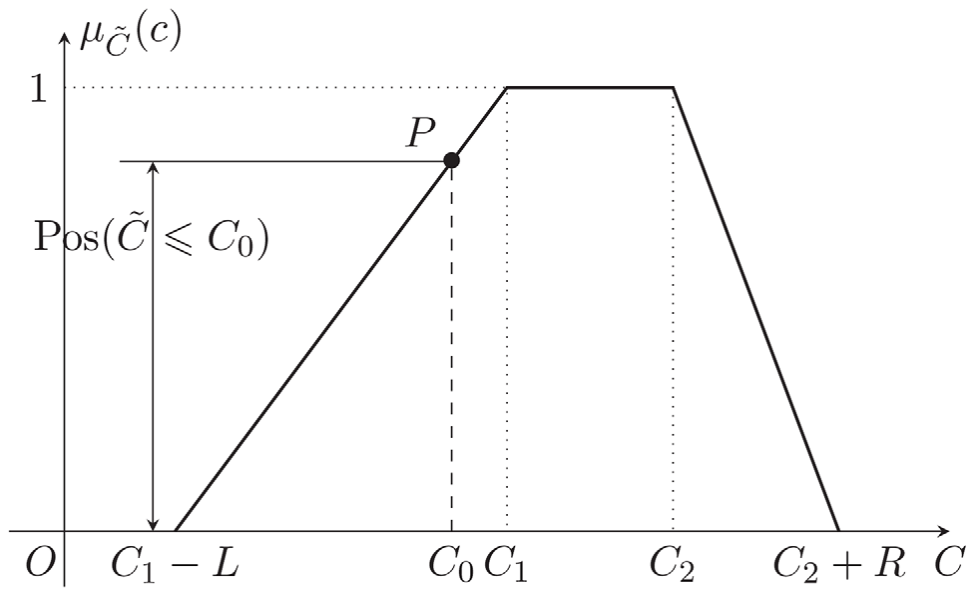
Treatment of objective: possibility-modality approach.

From Figure 7, we can get
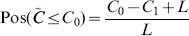



Hence, the objective of FTP can be transformed to
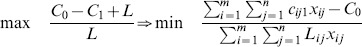
(19)


Such that the FTP turns to
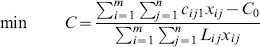


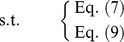



Again, this is a fractional programming problem which can be transformed to a linear programming problem by the substitution
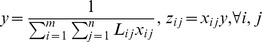



We obtain the following model.
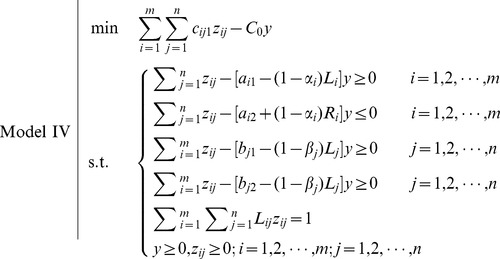



To sum up, from the view of possibility and necessity, and by fractile and modality approach, we proposed four ways to defuzzify FTP to four crisp linear programming problems. During the transformation process, some parameters are introduced to clarify the decision-maker's subjectiveness about fuzziness, which makes the solutions more practical. As for constraints, we introduced 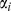 and 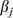 to reflect the decision-maker's requirement on the extent how the constraint is satisfied in the view of possibility. With respect to the objective function, in the fractile approach we proposed 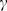 as the lower bound of necessity and 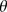 as the lower bound of possibility that the total cost is not larger than a given level set by the decision-maker. While in the modality approach, 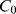 was introduced as the upper bound of total cost that decision-maker expected. In brief, these parameters reflect the decision-maker's attitude to the fuzziness in FTP from the view of possibility or necessity.

## Numerical example

In this section, numerical examples of FTP will be presented to demonstrate and verify the proposed approaches. All established models are originated from the above models based on the different parameter settings. All models are solved by the Lingo software, so the solving process is omitted for simplification. The description of transportation problem is standard tables, where the central part is the cost 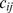, the column “Supply” are 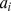 and the row “Demand” are 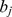. While in the solution tables, the central part is the quantities of transportation from 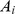 to 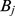.

**Figure 8:**
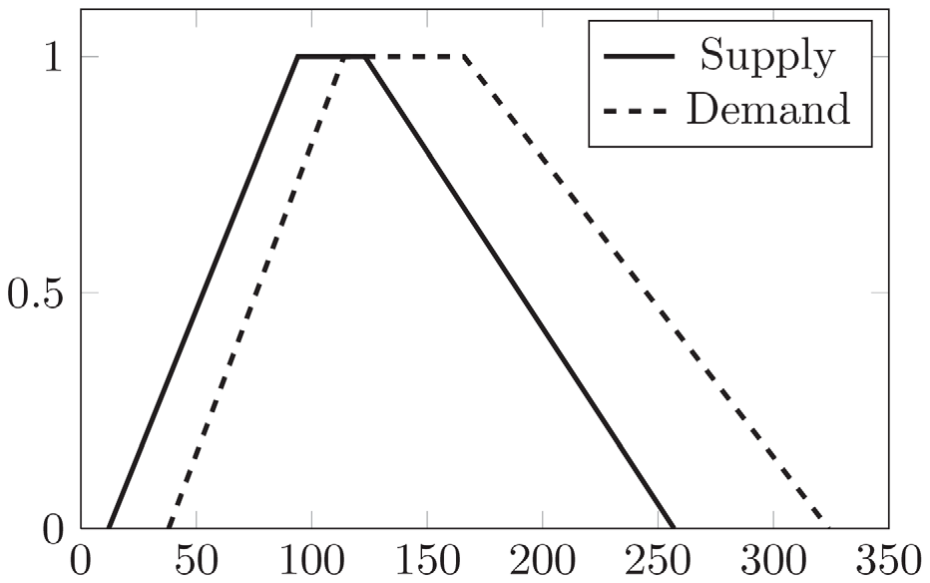
The relationship between supply and demand.

Table 1 is a FTP with four origins 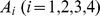 and five destinations 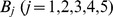 because the supply, demand and cost of unit are assumed to be fuzzy trapezoidal numbers. By adding up for origins' supply and five destinations' demand in Table 1, the total supply and demand are fuzzy trapezoidal numbers (94,123;82,134) and (114,166;76,158) depicted in the Figure 8.

**Table 1:** Data of the example.

	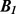	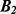	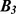	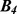	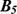	Sup.
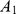	(8,9;3,2)	(2,3;1,5)	(2,5;1,6)	(10,16;4,8)	(7,12;5,5)	(22,28;20,30)
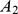	(3,6;1,8)	(8,11;4,3)	(12,16;5,8)	(3,5;1,6)	(2,6;1,7)	(28,35;25,36)
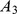	(13,16;5,8)	(3,8;1,10)	(10,18;4,9)	(4,10;1,1)	(12,20;5,2)	(16,24;12,28)
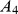	(18,23;3,6)	(12,18;5,9)	(11,15;4,8)	(30,36;6,4)	(6,9;2,1)	(28,36;25,40)
Dem.	(20,30;12,30)	(22,32;15,30)	(32,40;25,40)	(12,28;6,18)	(28,36;18,40)	

To solve this FTP, firstly we set the value of 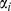, 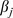, 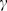 and 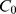 as follows:
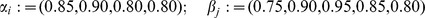


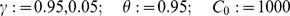



Then according to (7) and (9), the supply and demand constraints can be converted to different bounded intervals as shown in Table 2 based on the given value of 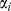 and 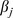.

**Table 2:** The crisp transportation problem originated from Model I.

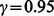	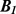	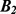	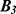	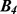	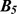	Sup.
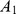	10.90	7.75	10.70	23.60	16.75	[19.0, 32.0]
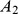	13.60	13.85	23.60	10.70	12.65	[25.5, 38.6]
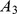	23.60	17.50	26.55	10.95	21.90	[13.6, 29.6]
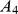	28.70	26.55	22.60	39.80	9.95	[23.0, 44.0]
Dem.	[17, 37.5]	[20.5, 35]	[30.75, 42]	[11.1, 30.7]	[24.4, 44]	

By using Model I, let 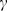 to be 0.95 and 0.05 respectively. And based on the Eq.(12), the cost of FTP can be transformed into a crisp value as shown in Table 2.

**Figure 9:**
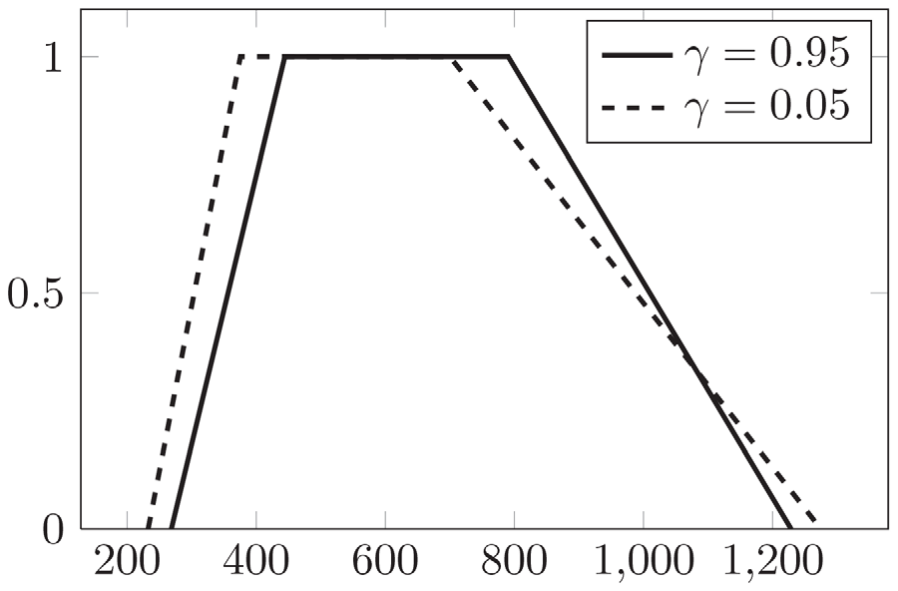
Fuzzy total cost when 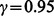 and 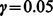.

Solve the two crisp transportation problems by linear programming, the optimal solution is in Table 3, and the fuzzy total cost is depicted in Figure 9 for comparative analysis.

**Table 3:** Solution from Model I (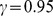).

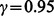								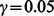						
	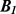	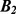	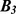	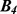	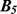	Supply	Pos	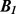	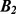	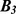	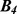	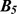	Sup.	Pos
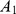	0	1.75	30.75	0	0	32.5	1.00	0	1.75	30.75	0	0	32.5	1.00
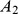	17	16.25	0	0	0	33.25	1.00	17	5.15	0	11.1	0.57	33.82	1.00
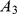	0	2.5	0	11.1	0	13.6	0.80	0	13.6	0	0	0	13.6	0.80
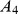	0	0	0	0	24.4	24.4	0.86	0	0	0	23.83	23.83	0.83	
Dem.	17	20.5	30.75	11.1	24.4	103.75		17	20.5	30.75	11.1	24.40	103.75	
Pos	0.75	0.90	0.95	0.85	0.80			0.75	0.90	0.95	0.85	0.80		
Total cost		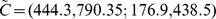						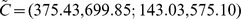						

From Table 3 and Figure 9, it can be found that the distribution varies with the changing of 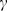. However, the total transportation, supply from different origins and demand to different destinations are same. And, the possibility requirement of supply and demand constraints are almost satisfied. From the point of fuzzy total cost, it gets smaller when 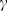 is smaller.

According to Model II, assume that the decision-maker expects that the lower bound of possibility that total cost is not larger than some given level 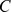 is 0.95, i.e., let 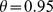. The solution is in the Table 4.

**Table 4:** Solution from Model II (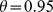).

	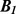	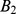	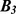	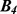	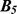	Sup.	Pos
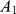	0	1.75	30.75	0	0	32.5	1.00
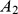	17	0	0	11.1	1.4	29.5	1.00
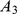	0	18.75	0	0	0	18.75	1.00
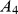	0	0	0	0	23	23	0.80
Dem.	17	20.5	30.75	11.1	24.4	103.75	
Pos	0.75	0.90	0.95	0.85	0.80		
Total	cost	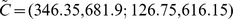					

In model III, let 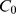 equals 1000, the solution is listed in Table 5.

**Table 5:** Solution from Model III (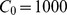).

	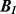	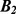	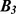	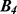	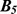	Sup.	Pos
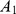	0	1.75	30.75	0	0	32.5	1.00
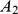	17	5.15	0	11.1	0	33.25	1.00
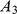	0	13.6	0	0	0	13.6	0.80
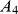	0	0	0	0	24.4	24.4	0.856
Dem.	17	20.5	30.75	11.1	24.4	103.75	
Pos	0.75	0.90	0.95	0.85	0.80		
Total	cost	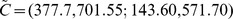					

From Table 5, we can easily get that
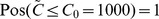



Hence, the possibility that the total cost is not larger than given 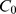 is satisfied. This is reasonable because 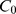 is set to be the upper bound of the minimal total cost which a decision-maker can afford.

In model IV, similarly let 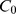 equals 1000, and solution is listed in Table 6. From Table 6, the following relationship exist.
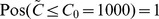



**Table 6:** Solution from Model IV (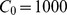).

	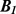	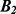	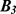	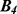	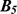	Sup.	Pos
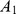	0	1.75	30.75	0	0	32.5	1.00
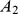	17	0	0	11.1	1.4	29.5	1.00
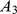	0	18.75	0	0	0	18.75	1.00
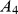	0	0	0	0	23	23	0.80
Dem.	17	20.5	30.75	11.1	24.4	103.75	
Pos	0.75	0.90	0.95	0.85	0.80		
Total	cost	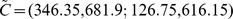					

Similar results can be achieved as shown in Model III.

## Conclusions

In this paper, a simple but effective parametric method was introduced to solve fuzzy transportation problem. By using possibility theory in fractile and modality approach, the fuzzy transportation problem is transformed into four types of crisp linear programming problems. In the process of transformation, some parameters are introduced to reflect decision-maker's attitude to the uncertainty or fuzziness. The methods proposed in this paper can be used for all kinds of fuzzy transportation problem, whether triangular and trapezoidal fuzzy numbers with normal or abnormal data.
